# Anti-aging: senolytics or gerostatics (unconventional view)

**DOI:** 10.18632/oncotarget.28049

**Published:** 2021-08-31

**Authors:** Mikhail V. Blagosklonny

**Affiliations:** ^1^Roswell Park Cancer Institute, Buffalo, NY 14263, USA

**Keywords:** geroscience, senolytics, hyperfunction theory, aging, sirolimus

## Abstract

Senolytics are basically anti-cancer drugs, repurposed to kill senescent cells selectively. It is even more difficult to selectively kill senescent cells than to kill cancer cells. Based on lessons of cancer therapy, here I suggest how to exploit oncogene-addiction and to combine drugs to achieve selectivity. However, even if selective senolytic combinations will be developed, there is little evidence that a few senescent cells are responsible for organismal aging. I also discuss gerostatics, such as rapamycin and other rapalogs, pan-mTOR inhibitors, dual PI3K/mTOR inhibitors, which inhibit growth- and aging-promoting pathways. Unlike senolytics, gerostatics do not kill cells but slow down cellular geroconversion to senescence. Numerous studies demonstrated that inhibition of the mTOR pathways by any means (genetic, pharmacological and dietary) extends lifespan. Currently, only two studies demonstrated that senolytics (fisetin and a combination Dasatinib plus Quercetin) extend lifespan in mice. These senolytics slightly inhibit the mTOR pathway. Thus, life extension by these senolytics can be explained by their slight rapamycin-like (gerostatic) effects.

## INTRODUCTION

Spiced up with words like “emerging” and “promising” [1–4], numerous excellent reviews on senolytics can be friendly parodied in one sentence: ‘New promising strategies to fight devastating diseases are rapidly emerging, fueling new hopes and promising healthier lifespan with potential benefits to win the war on aging by using emergent senomorphics and promising senolytics’.

Despite these promises, only two studies showed lifespan extension by senolytics in mammals. Namely, fisetin extended lifespan in a small mouse study [5]. A combination of Dasatinib plus Quercetin (D+Q) increased median lifespan from 937 days to 996 days (by 6.3%) in mice (see Figure 6I in ref. [6]). As we will discuss, this modest increase in lifespan can be explained not only by killing of senescent cells, but also by off-target effects such as mTOR inhibition. These senolytics are available for human use and, for reasons discussed elsewhere [7], can be used for life extension in humans without the need for lifelong clinical trials.

### Senolytics

The term senolytics, drugs that selectively kill senescent cells, was introduced by Kirkland and Tchkonia in 2015 [8]. Senolytics must extend lifespan by killing senescent cells, not by off-target mechanisms [8]. Kirkland and co-workers attempted to develop senolytics using bioinformatics followed by screening for siRNAs that kill senescent cells, followed by screening of potential drugs that may target these pathways [8]. They hypothesized that senescent cells can be selectively targeted, because they express pro-survival pathways, making them resistant to death [8–10]. While it seems paradoxical to kill cells, because they are resistant to killing there is a relevant analogy in oncology known as oncogene-addiction.

### Crossroad of oncology and geroscience

The field of senolytics is at a crossroads of two disciplines: oncology and gerontology. Development of drugs that kills senescent cells selectively is an oncology-like task. All potential senolytics are either approved for cancer therapy (dasatinib, venetoclax) or experimental anti-cancer drugs (Fisetin and Quercetin), including failed drugs (the Hsp-90 inhibitor geldanamycin). But whether killing of senescent cells is the goal of anti-aging therapy is the realm of gerontology.

### Lessons from cancer therapy

If cancer cells could be killed selectively without killing normal cells, then cancer would be curable. For almost a century, millions of scientists worldwide have worked on the cure for cancer, spending hundreds of billions of research dollars. Still, most common cancers remain incurable by chemotherapy. So, similarly, we cannot expect miracle from senolytics in such short time. Especially given that the cancer cell is an easier target than the senescent cell. In cancer therapy, some selectivity can be achieved by targeting cell proliferation. For example, microtubule active drugs such as paclitaxel and vinblastine kill cells entering mitosis. But targeting proliferation cannot be possibly exploited for killing senescent cells.

A second way to achieve selectivity in cancer therapy is targeting the tissue of cancer origin [11]. For example, targeting all prostate cells (normal and cancer) by anti-androgen deprivation or breast epithelial cells by anti-estrogens. This approach is not applicable for anti-aging therapy.

The third approach is targeting oncogenes that support the survival of cancer cells. For example, the Bcr-Abl oncoprotein, an anti-apoptotic kinase, drives chronic myelogenous leukemia [12]. Dasatinib, an inhibitor of Bcr-Abl, is approved for treatment of the BCR-ABL-driven leukemias [13].

### Oncogene addiction and matching targets (technical description)

Inhibitors of Bcr-Abl (imatinib and dasatinib) induce apoptosis in Bcr-Abl-expressing cells [12]. The paradox is that Bcr-Abl is not necessary for cell survival, if cells do not have it, but it becomes necessary, if cells do have it. Normally, no cell has Bcr-Abl. For example, HL60 leukemia cells do not have and do not need Bcr-Abl. Inhibitors of Bcr-Abl exert no effect on HL60 cells [14]. But once HL60 cells are transfected with Bcr-Abl, they become Bcr-Abl-addicted. Inhibitors of Bcr-Abl induce apoptosis in BCR-Abl-transfected HL60 cells, while they have no effect on parental HL60 cells [14]. And this is even more surprising because Bcr-Abl renders HL60 resistant to standard chemotherapy. Oncogene addiction can be explained by the dam model [15]. Because Bcr-Abl blocks the apoptotic cascade, another pro-survival mechanism (for example, Bcl-2) may become dispensable. Specifically, whereas parental HL60 cells express high levels of Bcl-2, Bcr-Abl-expressing cells have no Bcl-2 [16]. Due to loss of Bcl-2, caspase-9 is activated (Figure 2 in ref. [17]). However, this activation does not cause apoptosis due to the Bcr-Abl dam. When the Bcr-Abl dam is inactivated by dasatinib or degraded by geldanamycin, then the stream overflows, killing the cell [15]. Combined targeting of BCL-2 and BCR-ABL eradicates chronic myeloid leukemia stem cells [18].

Noticeably, these anti-cancer drugs developed for oncogene-addicted cancers were re-discovered as senolytics: the Bcr-Abl kinase inhibitor dasatinib, the Bcl-2/BclxL inhibitors Venetoclax (ABT-199) and Navitoclax (ABT-263) and Hsp-90 inhibitors (geldanamycin).

Another side of the same coin is synthetic lethality [19, 20]. In 1997, Synthetic lethality was defined as a condition when “the loss of either of two genes is viable for the cell, but the simultaneous inactivation of both genes is lethal” [21]. In other words, loss of one target renders cell sensitive to inhibition of its matching target.

**Figure 1:**
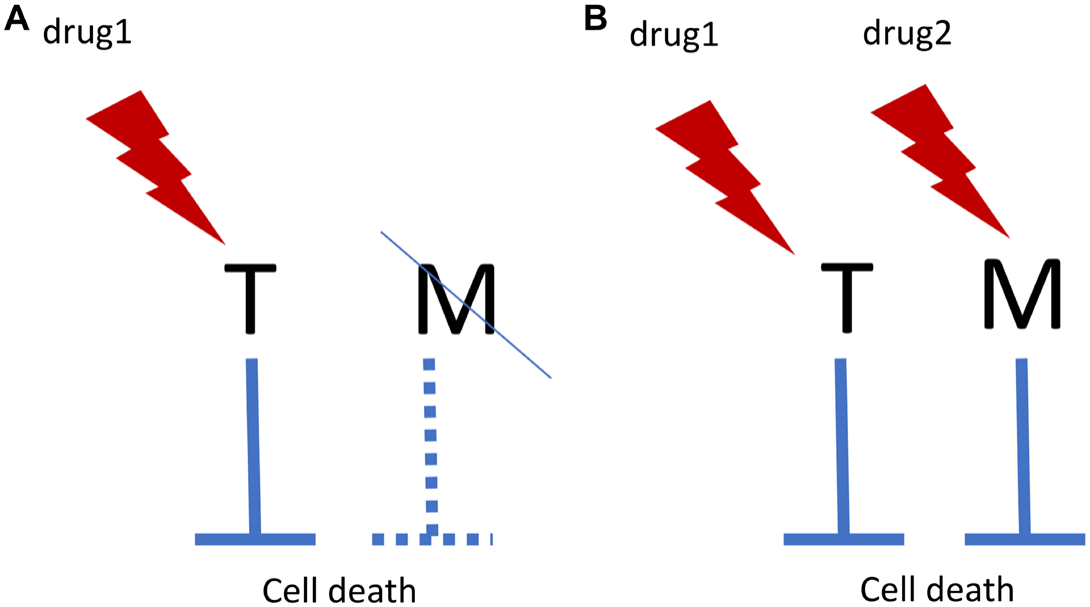
Oncogene addiction/synthetic lethality and matching targets. (**A**) In oncogene addiction, expression of an anti-apoptotic oncoprotein (Target, T) eventually leads to deactivation of matching (M) survival pathway. For example, T is Bcr-Abl and M is Bcl-2. Drug 1 kills such cells selectively. In synthetic lethality, loss of M renders cells sensitive to drug 1. (**B**) Matching drug combination. Targeting M by drug 2 renders cells sensitive to drug 1. And vice versa.

Let us take this one step further: combinations aimed at both targets (Figure 1). Matching drug combinations can selectively kill cells with known genetic/epigenetic background, while sparing other cells [22, 23]. I discussed anti-cancer combinations previously [23, 24]. And it is remarkable that out of two senolytic modalities that extend lifespan in mice, one is an empirical drug combination. Also, remarkably, one drug in this combination is the Bcr-Abl inhibitor that is used for oncogene-addicted leukemias. The next step would be designing mechanism-based combinations aimed toward matching and well-defined targets.

### Senolytics: from oncology back to gerontology

The main problem in cancer therapy is how to kill cells selectively. Senolytics face a similar problem. Venetoclax (ABT-199) and Navitoclax (ABT-263), inhibitors of Bcl-2 and BclxL, are approved as anti-leukemia drugs [25]. These drugs have serious side effects due to damage of neutrophils and blood platelets. HSP-90 inhibitors (e.g., geldanamycin), which target multiple oncogene-addiction [14, 26] were tested for cancer treatment but have not be approved because of their toxicity even at intermittent doses typical for cancer therapy.

But toxicity is not the only problem. In oncology, a cancer cell is the undisputed target, which must be killed or at least permanently arrested. But is a senescent cell the correct target to slow down organismal aging? [27, 28]. Do senescent cells drive aging, or they are just markers of aging? Is it feasible to kill senescent cells instead of rejuvenating them? And more fundamental questions: What is cellular senescence? Is it a loss of function? If yes, why then do we choose to decrease their functioning further by killing these cells? Or in contrast, is senescence a hyperfunction, such as the Senescence-Associated Secretory Phenotype (SASP), in which case it makes sense to kill these cells? Is senescence caused by damage? And if yes, some senolytics are damaging drugs and may cause senescence by themselves [29]. Or senescence is not functional decline due to accumulation of molecular damage? Then what causes cellular senescence and organismal aging?

### Senescence in cell culture

The program of cellular senescence consists of two steps: cell cycle arrest followed by gerogenic conversion from initially reversible arrest to senescence (geroconversion) [30, 31]. The cell cycle arrest can be induced by a variety of means: DNA damaging and anticancer drugs, telomere shortening, hyperactivation of oncogenic pathways (Ras, Raf, Akt) and ectopic expression of p21 and p16. In all these cases, arrest is ultimately mediated by p21 and p16, which inhibit CDK [30, 31].

When the cell cycle is arrested by p16 or p21, then growth-promoting pathways such as mTOR and MAPK convert this arrest to irreversible senescence (geroconversion). Cellular senescence is caused by geroconversion, not by cell cycle arrest. Geroconversion is a continuation of cellular growth, when actual growth is limited because of the cell cycle arrest [32]. Geroconversion is associated with the proliferation-like activity of mTOR and MAPK pathways. Geroconversion is a proliferative state of non-proliferating cells [30, 31]. Hyperfunctional growth-promoting pathways lead to cellular hypertrophy (large flat morphology), hyper-secretion (senescence-associated secretory phenotype, SASP) and lysosomal hyperfunction (senescence associated beta-galactosidase, SA-β-gal), accumulation of lipids (red-O-staining), overexpression of cyclin D1, hyperproduction of lactate, as well as secondary growth factor- and insulin-resistance [30, 31]. These are hallmarks of cellular senescence, predictable by the model that cellular senescence is a continuation of cellular growth [33]. When the cell gets arrested in the presence of rapamycin, geroconversion is decelerated [34]. Rapamycin maintain reversible quiescence (or G_0_), by delaying senescence. Rapamycin inhibits cellular growth in size and thus slows down geroconversion, which is a continuation of growth [30, 31].

### Geroconversion *in vivo*

In G_0_/quiescent cells, mTOR is inactive. Then activation of mTOR leads either to proliferation or to geroconversion [35]. In the organism, mTOR activation may lead to partial geroconversion such as the transition of stem cells from G_0_ to G_Alert_, associated with cell size growth [36]. Prolonged G_Alert_ leads to stem cell depletion [37]. Alternatively, quiescent stem cells may undergo geroconversion to senescence [38, 39]. mTOR is involved in stem cell senescence, and inhibition of mTOR maintains stem cell quiescence [37, 40–42].

### Senescent and gerogenic cells in the organism

**Figure 2:**
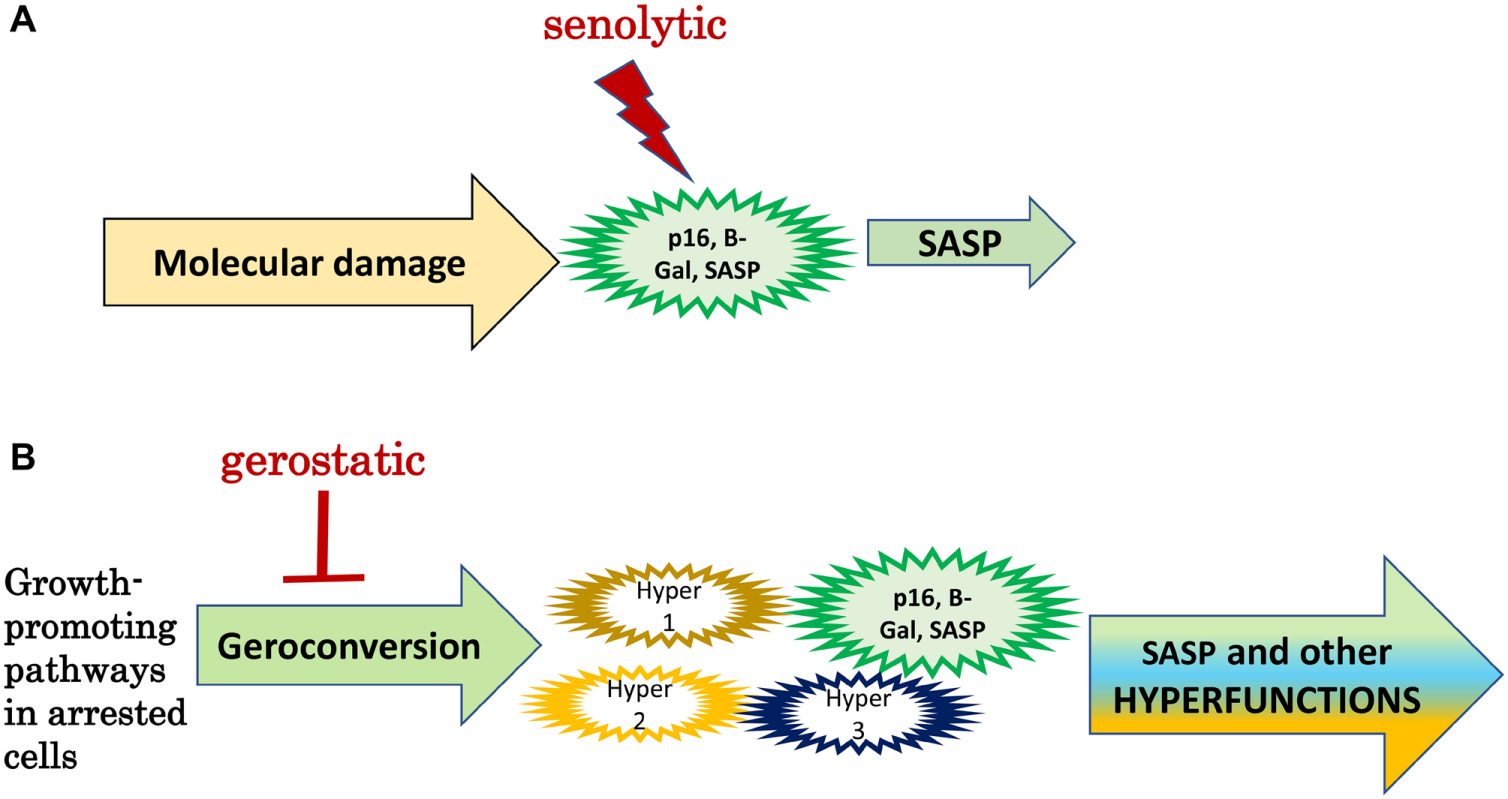
Senolytics versus gerostatics. (**A**) Senolytics: Standard model. Molecular damage causes functional decline associated with p16 expression, SA-β-gal-staining and SASP (a large green cell). SASP is involved in some diseases of aging. Senolytics kill senescent cells. (**B**) Gerostatics: simplified hyperfunction model. In arrested cells, growth-promoting and nutrient-sensing signaling pathways drive geroconversion instead of growth, rendering them gerogenic. Only a few cells (green) become phenotypically senescent. Most gerogenic cells are just slightly hyperfunctional (hyper 1, 2, 3). SASP is one of numerous hyperfunctions. Activated p16-positive macrophages are an example of gerogenic cells. Hyperfunctional cells drive age-related disease and aging is a sum of all diseases.

According to mainstream theories of aging, cellular senescence is a permanent growth arrest caused by DNA damage and other stresses. SASP promotes organismal aging and its diseases (Figure 2A). By killing senescent cells, senolytics delay diseases and/or aging [43–45].

According to hyperfunction theory, cellular senescence is a continuation of cellular growth and cellular functions, leading to hyperfunctions [46]. SASP is only one of numerous hyperfunctions, which are tissue-specific (cells of different tissues have different functions). Although noticeable, fully senescent cells are rare in the organism. According to hyperfunction theory (Figure 2B), most cells undergo partial geroconversion, but only some cells (mostly of connective tissue and macrophages) acquire classically senescent morphology. Most cells undergo partial geroconversion (or no geroconversion at all). According to hyperfunction theory, the key feature of senescent cells is hyperfunction caused by higher than optimal activity of signaling pathways such as mTOR. These pathways drive development and growth but are not deactivated enough in post-development [46]. Hyperfunctional cells are gerogenic, producing age-related diseases. Senescent cells with p16 and SA-β-gal expression are a subgroup of gerogenic cells.

P16 is a marker of cell cycle arrest, but cell cycle arrest is not yet senescence. SA-β-gal is a hallmark of hyperfunctional lysosomes [47–49]. Cells arrested by serum-starvation and by contact-inhibition are also SA-β-gal-positive [47], (Figure 4 in [50]).

Hyperfunction theory is based on the cell culture model of proliferation-like level of signaling pathways in non-proliferating cells. This is the simplest hyperfunction. Quasi-programmed nature of aging is not an absolutely essential element of hyperfunction theory.

### Non-senescent cells in organismal aging

According to hyperfunction theory, phenotypically-senescent cells are a subgroup of gerogenic cells. The fully senescent phenotype develops when growth-promoting pathways (for example, mTOR, MAPK) are active in acutely arrested (by DNA damage, for instance) cells [31]. Some other gerogenic cells are the product of partial geroconversion. And some gerogenic cells are not necessarily different from young, normal cells; it is enough that their function is not sufficiently decreased, when it becomes unnecessary in post-development. For example, cells that facilitate collagen cross-linking (an important function in development), should not do that in post-development (except in special cases, such as wound healing [51]. Or, the nematode *Caenorhabditis elegans* senesces without senescent cells. Simply, cells continue their developmental and reproductive functions in post-development and thus drive quasi-programmed (age-related) diseases [52, 53]. For example, they continue to produce yolk when it is not needed anymore, leading to intestinal atrophy and ectopic yolk deposition [54]. As another example, teratoma-like tumors develop from unfertilized oocytes which enter the uterus and become hypertrophic after exhaustion of sperm stocks [55, 56].

I believe that phenotypically-senescent cells contribute to some age-related diseases in some (but not all) organisms. Aging is driven by all gerogenic cells combined (Figure 2B).

### Gerostatics in life extension

A decade ago, I introduced the term gerostatic or gero-suppressant (see for references [30, 31]). The immuno-*suppressant* rapamycin is a prototypical gero-*suppresssant* (gerostatic). The term gerostatic emphasizes static effects of rapamycin on both proliferation and geroconversion. At low doses, inhibitors of the mTOR kinase [57–59], PI3K and MEK [60, 61], S6K [61], PDK1 [62] and mdm-2, such as nutlin-3B [63, 64] are gerostatics. Deep hypoxia [65] and contact inhibition [50] are physiological gerostatics. In contrast, metformin is not gerostatic because it does not affect geroconversion and cellular senescence. Gerostatics should not be confused with senostatics. The term gerostatic has precise meaning: a drug that slows down geroconversion.

Like cellular senescence is a continuation of cellular mass growth [46], organismal aging is a continuation of developmental growth, driven in part by growth-promoting pathways [46, 52, 53, 55, 56, 66, 67]. Signal pathways that drive geroconversion in cell culture also promote aging in animals. Inhibition of the IGF-1/PI3K/mTOR/S6K pathway delays senescence and increases lifespan in animals including mammals. For example, mice with reduced mTOR expression [68], low mTORC1 activity due to growth hormone resistance [69, 70] are small and live longer [68–70].

As a gerostatic, rapamycin suppresses growth and senescence in yeast [71] and mammalian cells [32, 40–42, 72–82]. Rapamycin slows aging, stem cell exhaustion and extends lifespan in the simplest organism: *Hydra* [83]. Rapamycin extends lifespan in *C. elegans* [84] and *Drosophila* [85, 86]. Rapamycin increases lifespan and healthspan in mice [42, 87–121].

### Fisetin inhibits the PI3K/mTOR pathway

Fisetin (3, 7, 3′ ,4′-tetrahydroxyflavone) inhibits multiple signaling kinases, including the PI3K/mTOR pathway and is considered a natural dual inhibitor of PI3K/Akt and mTOR signaling [122–131]. Fisetin inhibits the mTOR pathway both indirectly and directly by binding to mTOR and its downstream target, p70S6K [129]. Fisetin causes death of cancer cells, which is associated with mTOR inhibition [124–129]. Fisetin exerts multiple rapamycin-like effects in animals. It prevents cardiac hypertrophy by inhibiting mTOR [131]. Fisetin inhibits Akt, S6K1 and mTORC1, S6K1 in adipose tissue and prevents adipocyte differentiation and obesity in HFD-fed mice [130].

### Quercetin inhibits multiple kinases

In numerous studies, quercetin inhibited the PI3K/Akt/mTOR pathway by multiple mechanisms in cell culture and animals [132–143]. At concentrations that also inhibit the PI3K/Akt/mTOR-signaling pathway, quercetin suppresses cancer cell growth [137–138]. Quercetin inhibits multiple kinases including ABL1, Aurora-A, -B, -C, CLK1, FLT3, JAK3, MET, NEK4, NEK9, PAK3, PIM1, RET, FGF-R2, PDGF and may kill cells in mitosis [144]. Inhibition of multiple targets, when only one is an intended target, may increase side effects without increasing therapeutic effect.

### Dasatinib and Quercetin (D+Q) combination

Quercetin alone does not extend lifespan in mice [145], but a Dasatinib and Quercetin (D+Q) combination extended lifespan. The first empirical senolytic combination includes D, originally developed to target oncogene-addiction in leukemia, and Q, which inhibits the mTOR pathway, among numerous others. Dasatinib is an inhibitor of multiple tyrosine kinases including Bcr-Abl, ABL, SRC, c-KIT, PDGFR and ephrin receptor. Due to its inhibition of multiple kinases, it suppresses bone marrow, resulting in pancytopenia [13] and causing pulmonary endothelial cell apoptosis, lung vascular toxicity, pleural effusions and predisposition to pulmonary hypertension [146]. As a long-term side effect, Dasatinib increases mortality from ischemic heart disease [147, 148].

In humans, D 100 mg and Q 1000 mg given for three days decreased the number of p16- and SA-β-gal-positive cells in adipose tissue [9]. In patients with idiopathic pulmonary fibrosis, the senolytics effect of this treatment on relevant markers was inconclusive [149].

However, Kovacovicova et al. found that D+Q was ineffective in clearing chemotherapy-induced senescent cells. Furthermore, D+Q exerted acute pro-tumorigenic effects [150]. And furthermore, dasatinib plus quercetin treatment led to exacerbation of obesity- and age-dependent liver disease progression [151].

### Do senolytics exist?

By the strict definition given by Kirkland [8], the existence of senolytics has not yet been proven. Although F and D+Q decrease the number of SA-β-gal and p16-positive cells in some tissues, there is no proof that this decrease is due to the killing of senescent cells in the organism. It could be due to reduction of these markers per cell, or even cell rejuvenation. In fact, rapamycin, which does not kill senescent cells, decreases expression of SA-β-gal and p16 [73, 74, 152]. In the organism, low doses of rapamycin decrease levels of p16 and tend to decrease SA-β-gal activity [153]. Given that current senolytics (F, D+Q) can inhibit mTOR, this scenario is possible. In order to demonstrate that senolytics work as senolytics, it is necessary to detect dead and apoptotic senescent cells, rather than only a decrease in SA-β-gal and p16. This is exactly how cytotoxic therapy is validated in oncology [154–156].

One may argue that because senolytics can be administered intermittently—a ‘hit-and-run’ approach, rather than continuously (daily)—this proves that they kill cells. This argument is not compelling. For example, rapamycin (a gerostatic, which does not kill cells) nevertheless can be given intermittently and transiently to extends lifespan and prevent cancer [88, 104, 111, 112, 157–160]. Even a single dose has long lasting effects. For example, a single administration decreases weight gain for at least 10 weeks, by shifting the set point long-term [161]. Rapamycin treatment for 2 weeks in young mice results in long-term preservation of primordial follicles and prolongation of ovarian lifespan in old mice [162].

Hyperfunctional senescent cells over-secrete cytokines and growth factors that may drive senescence of other cells and make them hyperfunctional too. Mutual overstimulation establishes self-maintained positive feedback loops. I suggest that disruption of such loops, even by a single (but high) dose of rapamycin, can have prolonged effects without killing cells.

SA-β-gal-positive/p16-expressing cells are not always senescent [163–166]. SA-β-gal and p16 can be reversibly induced in macrophages by physiological stimuli [163–166]. In groundbreaking studies, Gudkov and co-workers found that “significant proportion of p16/βGal-positive cells in aging mice are activated macrophages” [163–165]. Given that activated (hyperfunctional) macrophages and macrophage-derived foam cells are involved in age-related diseases, this may explain why the elimination of p16/ SA-β-gal-positive positive cells can be beneficial.

Although hyperfunction is a characteristic of senescent phenotype, p16/ SA-β-gal-positive macrophages are different from senescent cells used to screen for senolytics in cell culture [163–165].

Activated macrophages are gerogenic. Oxidized Low-Density Lipoprotein (ox-LDL) activates macrophages and induces formation of foam senescent cells characterized with SA-β-gal and p16 expression [167]. Remarkably, Quercetin [167] and Fisetin [168] inhibit formation of foam cells, prevent SA-β-gal and p16 induction and delay senescence [168].

**Figure 3:**
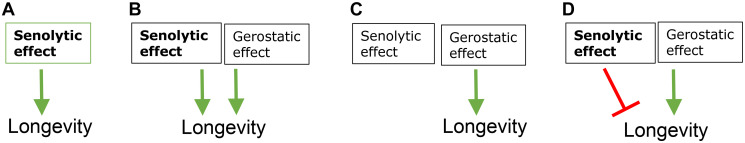
Potential mechanisms of life-extensions by fisetin and D+Q. (**A**) Life extension is purely through their senolytic effects. (**B**) Senolytic and gerostatic (off target) effects are additive (**C**) Senolytic effect is either absent or irrelevant. Life extension is purely through gerostatic (off target) mechanism. (**D**) Senolytic effect is detrimental and antagonizes life extension. Green arrows - stimulation; red symbol - inhibition.

Given that current senolytics may work as gerostatics, the significance of killing of senescent cells is unclear, even if it occurs (Figure 3). Is it the mechanism of life-extension or an unwanted side effect? Detrimental killing of senescent cells has been discussed in ref. [169].

### Two gerostatics as one senolytic

At low concentrations, inhibitors of MEK, PI3K, the mTOR kinase are gerostatics. At high concentrations, they may become cytotoxic, probably due to inhibition of multiple kinases (an off-target effect). (In contrast, rapamycin and other rapalogs are not cytotoxic at any achievable doses. Still, everolimus and rapamycin potentiate cytotoxicity of dasatinib against cancer cells [170, 171]). A combination of two gerostatics can act as a senolytic. For example, MEK inhibitors especially combined with pan-mTOR inhibitors are cytotoxic to some senescent cells [172, 173]. It would be important to investigate life-extension in mice by combinations of MEK and pan-TOR inhibitors, pan-mTOR inhibitors and rapamycin, MEK inhibitors and rapamycin.

## CONCLUSIONS

Rapamycin and other gerostatics do not kill senescent cells but slow down cell growth, gerogenesis and oncogenesis. Gerostatics mostly act on non-senescent cells, decreasing their hyperfunction and decelerating their geroconversion to senescence. Rapamycin robustly extends lifespan and tumor-free survival in mice. It is also effective, when used intermittently and transiently. In theory, inhibition of the mTOR pathway can explain life extension by current senolytics such as F, D+Q. However, it is not clear whether these senolytics inhibit mTOR sufficiently to slow aging at doses that are achievable in humans.

It is expected that rapamycin-like effect may be responsible for the therapeutic effects of senolytics in disease. Some senolytics are investigated for treatment of diseases such as idiopathic pulmonary fibrosis [10, 45, 149, 174]. Although the treatment of specific diseases is very important, it is a different story entirely. For example, DNA damaging drugs such as doxorubicin are successfully used for cancer therapy; insulin is a life-saving drug in terminal diabetes; glucocorticoids are useful for arthritis; antibiotics cure bacterial infections common in the elderly. And these conditions are common age-related diseases. But doxorubicin, insulin, corticosteroids and penicillin are not anti-aging drugs. And they do not extend lifespan in mice. Unless drugs extend lifespan, they are not drugs to treat aging as a common cause of age-related diseases. Life extension in mice by D+Q and F was shown in one study for each of these modalities [5, 6]. It is desirably to reproduce these results, preferably in a variety of mouse models, in order to advocate their use (alone or in combination with rapamycin) in humans for longer and healthier life. Given that these senolytics are available for human use and well-tolerated, they could be used under doctor supervision without life-long clinical trials [7]. But first it must be shown reproducibly that they extend lifespan consistently in animals.
